# Knockdown of BCL6 Inhibited Malignant Phenotype and Enhanced Sensitivity of Glioblastoma Cells to TMZ through AKT Pathway

**DOI:** 10.1155/2018/6953506

**Published:** 2018-10-18

**Authors:** Wen Song, Zhenling Wang, Pengcheng Kan, Zhuolin Ma, Yaru Wang, Qiaoli Wu, Xiuhua Yao, Biao Zhang

**Affiliations:** ^1^The Graduate School, Tianjin Medical University, Tianjin 300070, China; ^2^Tianjin Neurosurgery Institute, Tianjin Cerebral Vascular and Neural Degenerative Disease Key Laboratory, Tianjin Huanhu Hospital, Tianjin 300350, China

## Abstract

**Background:**

BCL6 was a critical prooncogene of human B-cell lymphomas which promoted tumor progress and contributed to malignant behavior in several kinds of cancers. This study was to detect the expression of BCL6 and its biological effect on glioma.

**Methods:**

RT-PCR and Western blot were used to detect the expression of BCL6 mRNA and protein in tissues and glioblastoma cell lines. The expression of BCL6 was knockdown in two glioblastoma cell lines (U87 and U251) using BCL6 shRNA. The CCK8, colony-formation, flow cytometry, Transwell, and wound-healing assays were used to evaluate the malignant phenotypic change of glioblastoma cells.

**Results:**

The expression of BCL6 was higher in glioma tissues and glioblastoma cell lines than normal tissues. Knockdown of BCL6 expression reduced the proliferation, migration, and invasion of glioblastoma cells. Moreover, knockdown of BCL6 changed expression of proteins related to malignant behaviors of glioblastoma cells. The suppression of BCL6 could increase chemosensitivity of U87 and U251 to temozolomide. Downregulation of BCL6 levels suppressed the expression of BCL2, cyclin D1, MMP2, and MMP9 proteins as well as two classic signaling pathway proteins p-AKT and p-ERK. Simultaneously, BAX and p21 protein levels were upregulated along with knockdown of BCL6.

**Conclusions:**

Our results indicated that BCL6 may be a tumor oncogene involved in the progression of glioma via affecting AKT and MAPK signaling pathways.

## 1. Introduction

Glioblastoma (GBM) is the most aggressive and lethal brain malignancy which is commonly referred to grade IV astrocytoma classified by World Health Organization (WHO). Despite there have great advancement in radiotherapy, chemotherapy, and surgical treatment, the median survival of GBM patients is merely about 14 months [1]. Glioblastoma is an incurable tumor with inevitable recurrence due to uncontrolled cellular proliferation, strong resistance to apoptosis, highly cellular invasion, and genomic alteration [2]. Moreover, the highly invasion of tumors cells are responsible for recurrence of GBM by evading surgical resection and resisting radiation and temozolomide (TMZ) [3]. Therefore, a great number of researchers have carried out investigation in order to make clear of the mechanism of glioblastoma over the years [1].

B-cell lymphoma 6 (BCL6), one of zinc finger transcriptional factor, works as a critical regulator in germinal center response. And this gene is also a key prooncogene of human B-cell lymphomas which participants in regulating the cell proliferation, differentiation and apoptosis of B and T cells [4]. Due to the mutation of BCL6 promotor, the overexpression of this gene was frequently found in lymphoma especially in the diffuse large B-cell lymphoma (DLBCL) [5]. Except for malignancy in the lymphoid system, accumulated evidence suggested that overexpression of BCL6 could regulate the progression of various human cancers including gastric cancer, breast cancer, ovarian carcinoma, and GBM [6–9]. The high expression of BCL6 in breast cancer cells promoted cell proliferation, migration, and invasion and indicated survival poor prognosis in both vitro and xenografts models [8, 10]. And in ovarian carcinoma, BCL6 facilitated the proliferation and invasion of tumor cells while its expression level tightly associated with the Federation of Gynecology and Obstetrics (FIGO) stage [7]. BCL6 was also reported to play an oncogenicity role in cerebral tumors. The overexpression of this gene was correlated with poor survival of the patients with neuroblastoma [11], and another study suggested that frequent translocation of BCL6 could induce the overexpression of BCL6 and inhibit the apoptosis of glioma cells. [12]. Similarly, Liang et al. found that BCL6 was an essential factor for glioma cells growth and its overexpression indicated poor prognosis of patients. In addition, they also explored several molecules related to proliferation of glioma [9]. However, whether the high expression of BCL6 in glioma is associated with invasion and chemosensitivity remains unclear. And as shown in previous studies, BCL6 is implicated in regulating multiple molecules that involved in malignant phenotype of cancers [8, 12, 13].

In this study, we identified the high expression of BCL6 in glioma tissues and cell lines, and then we investigated the role of BCL6 expression in regulation of glioblastoma proliferation, migration, invasion, and chemosensitivity in vitro. In addition, we explored the underlying molecular events of BCL6 action in glioblastoma cells.

## 2. Materials and Methods

### 2.1. Glioma Tissues and Nonmalignant Brain Tissues

In this study, we collected twelve glioma tissue samples including six high-grade gliomas and six low-grade gliomas, and six nonmalignant brain tissues were obtained from Tianjin Huanhu Hospital. The six nonmalignant brain tissues which obtained from patients with traumatic brain injury were used as control. A protocol to use patient samples was approved by the ethics committee of Tianjin Huanhu Hospital and informed consent was obtained from each patient according to the Declaration of Helsinki.

### 2.2. Cell Culture and Cell Transfection

Human GBM U87, A172, SNB19, U251, LN229, and LN308 cell lines were obtained from the Institute of Biochemistry and Cell Biology, Chinese Academy of Sciences, Shanghai, China. Dulbecco's modified Eagle's medium (DMEM, Gibco, USA) containing 10% fetal bovine serum (FBS, Gibco, USA) was used to incubate the cells, and the solutions were placed into a cell incubator for culture at 37°C with 5% CO2.

All plasmids were obtained from Gima Biol Engineering Inc. (Shanghai, China). Plasmid vectors pGPU6/GFP/NEO-shBCL6 contained a specific shRNA sequence according to the manufacture's protocol. The pGPU6/GFP/Neo encoding nonspecific sequences were used as negative controls. And cells were transfected with plasmid vectors mediated by Lipofectamine 2000 (Invitrogen, USA) following the manufacturer's directions.

### 2.3. mRNA Extraction and RT-PCR

Total RNA was extracted from tissue samples and glioblastoma cell lines with Trizol (Invitrogen, California, USA) according to the manufacturer's instructions. The total RNA was reverse-transcribed by cDNA Reverse Transcription Kit (Invitrogen, California, USA) following the manufacturer's protocol to get cDNA. To quantify expression of BCL6 mRNA, we used the Power SYBR green PCR master mix (Applied Biosystems, Carlsbad, USA) to detect BCL6 mRNA levels with a LightCycler 480 II PCR machine (Roche). GAPDH was measured as an internal control. The following primer pairs were used in this study:

  BCL6: 5'-CCAGCCACAAGACCGTCCAT-3' (forward)5'-CTCCGCAGGTTTCGCATTT-3' (reverse);
  GAPDH: 5'-TGCACCACCAACTGCTTAGC-3'(forward)5'GGCATGGACTGTGGTCATGAG-3' (reverse).

### 2.4. Cell Viability, Proliferation, and Colony-Formation Assay

Cell viability and proliferation were measured by CCK8 kit and colony-formation assay was performed. U87 and U251 cells were seeded in 96-well plates and cultured with DMEM medium (100*μ*L) containing 10% FBS overnight and then cells were transfected with shBCL6 or scrambled shRNA. At 24h, 48h, and 72h, the CCK8 regent was added to each well of 96-well plates and incubated at 37°C for 2h. The absorbance at 490 nm was measured using microplate reader and the background control was used as a blank. At 24h after transfection, cells were seeded in 12-wells at 100 cells per well and cultured with DMEM medium (1mL) containing 10% FBS which replaced every 3 days. And the cells were grown for about 14 days form visible colonies which stained with 0.1% crystal violet and imaged at a microscope. All experiments were performed in triplicate and repeated thrice.

### 2.5. Flow Cytometry Analysis

Cell apoptosis assays and cell cycle assays were performed at 48h after transfection. For cell cycle assays, 1 × 10^6^ cells were collected at 48h after transfection and then fixed them in 70% ice-cold ethanol overnight. Fixed cells were washed by PBS twice before stained with propidium iodide (PI, Calbiochem) for 20 minutes. Cell apoptosis was assessed by Annexin V-FITC/PI Apoptosis Detection kit (WanleiBio, Shenyang, China) according to the manufacturer's instructions. All the stained cells were analyzed on a FACSCantoII flow cytometry (BD Biosciences, USA) and analyzed by analysis software (FlowJo). Each experiment was performed in triplicate and repeated at least thrice.

### 2.6. Protein Extraction and Western Blot

For glioma tissues, a protein extract reagent (Bioteke Corporation, Beijing, China) was used to extract the protein following the manufacturer's directions. Cells seeded on six-well plates were washed with ice-cold PBS buffer and then homogenized by RIPA buffer supplemented with 1% protease inhibitors (Roche, Basel, Switzerland). After centrifugation, BCA Protein Assay Kit (Beijing Solarbio Science & Technology Co., Ltd., Beijing, China) was used to determine the concentration of supernatant protein. And then the isolated supernatant protein was added with sample buffer and boiled at 95°C for 5 min.

Total proteins were separated by SDS-PAGE at 80V for 3h and then transferred to a PVDF membrane (Millipore, Bedford, MA, USA) for another 2h. Afterwards, the membranes were blocked by 5% skim milk in TBST buffer for 2h and incubated with primary antibodies at 4°C overnight. The primary antibodies included anti-BCL6, anti-BCL2, anti-Bax, anti-cyclin D1, anti-p21, anti-MMP2, anti-MMP9, anti-AKT, anti-p-AKT, anti-ERK, anti-p-ERK, and anti-GAPDH. (CST, Massachusetts, USA). Membranes were then washed by 1% TBST for three times and incubated with secondary antibodies for 1h. Finally, proteins were visualized by the ECL procedure (Millipore, Bedford, MA, USA) and were analyzed by Image J software to get the gray intensity of bands. Each experiment was performed in triplicate and repeated at least thrice.

### 2.7. Transwell Migration and Invasion Assays

We used Transwell (Corning, Cambridge, USA) to determine the effect of BCL6 on glioblastoma cells migration and invasion in vitro. To evaluate the invasive capacity of cells, Matrigel (BD Biosciences, FranklinLakes, NJ, USA) was used to cover the membrane to form a matrix barrier. At 24h after transfection, cells were seeded on the upper chamber with or without a Matrigel-coated membrane at a density of 5.0 × 10^3^ cells/well in serum-free medium. The lower chamber was filled with 10% FBS as the chemoattractant. After 24 h incubation at 37°C, cells on the upper surface of the membrane were removed by wiping with a cotton swab, and cells on the lower surface were fixed with ethanol and stained with 1% crystal violet (Sigma-Aldrich, St Louis, MO, USA). And then the staining cells were photographed at 200× under a Nikon-TE2000 U inverted fluorescence microscope and counted by ImageJ software.

### 2.8. Wound-Healing Assay

Wound-healing assay was preformed to assess capacity for tumor cell motility. Transfected cells were seeded into 6-well plates and grow to reach confluence. And then the cell layer was scratched using a plastic tip and incubated in DMEM with 10% FBS at 37°C for 72 h. Photos were taken at 0, 24, 48, and 72 h under microscope to measure the wound-healing after scratched. The wound gap percentage was determined by the ratio of gap width at each time point to the wound width at 0 h. Each experiment was carried out in triplicate, and three random fields of each well were recorded.

### 2.9. Chemosensitivity Assay to Temozolomide Treatment

U87 and U251 cells were seeded in 96-wells plate overnight and transfected with scramble or shBCL6, and then treated with TMZ (Sigma, St Louis, USA) in different concentrations for 48h. CCK8 assay was performed to measure the cell survival. In addition, cell viability was measured at 0, 24, 48, and 72 h after TMZ (150*μ*M) treatment. To further investigate the effect of BCL6 in Chemosensitivity of TMZ, cells were divided into five group: (1) Scrambled group (cells were transfected with scrambled shRNA); (2) TMZ group (cells were treated with TMZ alone); (3) Scrambled + TMZ group(cells were transfected with scrambled shRNA and treated with TMZ); (4) shBCL6 group (cells were transfected with shBCL6); (5) shBCL6 + TMZ group (cells were transfected with shBCL6 and treated with TMZ). The apoptosis rate of each group was measured by flow cytometry assay.

### 2.10. Statistical Analyses

All data are presented as the mean ± standard deviation (SD) values and were analyzed with the GraphPad Prism 6.0 (CA, USA). One-way ANOVA or Student's t-test was used for Statistical analyses. A p value of less than 0.05 was considered statistically significant.

## 3. Results

### 3.1. Higher BCL6 Expresses in Glioma

**Figure 1:**
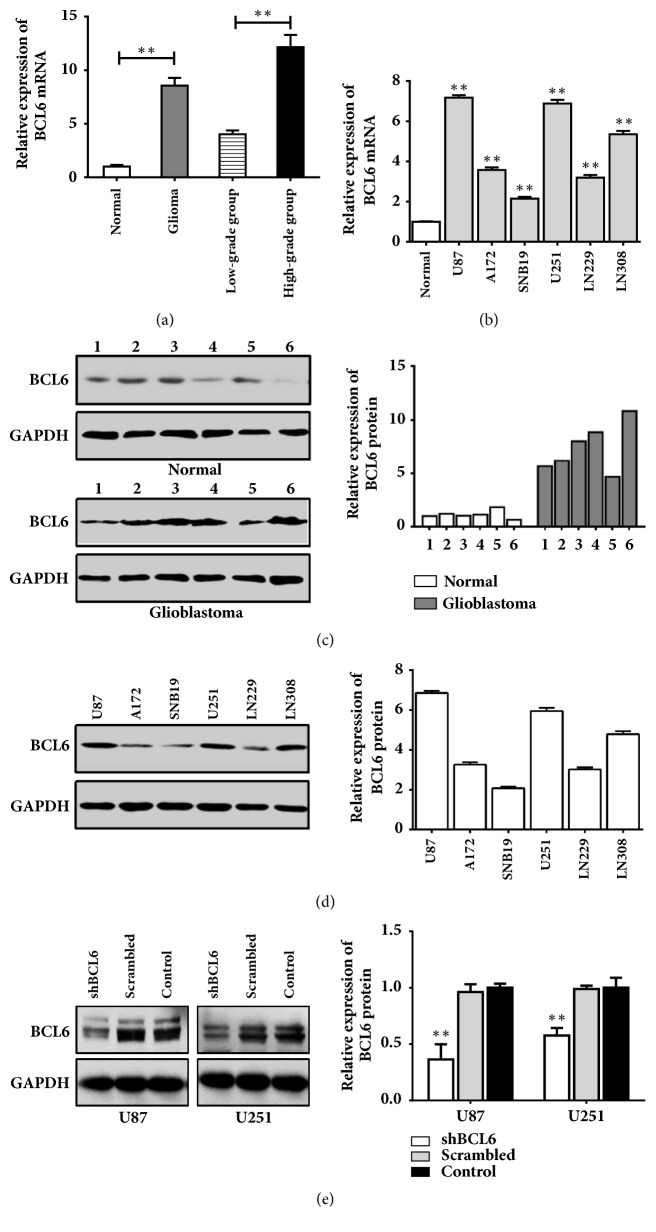
Expression of BCL6 mRNA and protein in human glioma tissues and glioblastoma cell lines. (a) The relative expression of BCL6 mRNA in 6 nonmalignant brain tissues and 12 glioma tissues was analyzed by RT-PCR. Data were normalized to the mean of mRNA in normal tissues. (b) The relative expression of BCL6 mRNA in glioblastoma cell lines (U87, A172, SNB19, U251, LN229, and LN308) was analyzed by RT-PCR. Data were normalized to the mean of mRNA in normal tissues. (c) The relative expression of BCL6 protein in nonmalignant brain tissues and glioma tissues was analyzed by western blot. Relative expression was calculated with respect to the first normal tissue. (d) The relative expression of BCL6 protein in glioblastoma cell lines by western blot. Relative expression was calculated with respect to the mean of protein in normal tissues. (e) Transfection efficiency of shBCL6 plasmid was confirmed by real-time PCR and western blot U87 and U251 cells. Relative expression was calculated with respect to the control group without treatment (*∗∗* p<0.01; *∗* p<0.05).

To identify the expression of BCL6 in glioma, we detected its levels in tissues and cell lines. The BCL6 expression in glioma was higher than in normal brain tissues, and the mRNA level of BCL6 significantly elevated in the high-grade glioma samples compared with the low-grade samples (Figure 1(a)). The glioma tissues also showed a higher level of BCL6 protein than the normal brain tissues (Figure 1(c)). Parallel to tissues, the six glioblastoma cell lines displayed a higher level of BCL6 in mRNA compared with normal brain tissues (Figure 1(b)). And BCL6 was expressed to different in glioblastoma cell lines (Figure 1(d)).

To investigate the role of BCL6 in progress of malignant gliomas, we reduced BCL6 expression by transfecting shBCL6 plasmid into the human glioma cell lines U87 and U251. Transfection efficiency was confirmed by real-time PCR and western blot analysis of BCL6 levels in the transfected glioma cells. BCL6 protein was decreased by 59.7% in U87 cells and 41.3% in U251 cells which were transfected with shBCL6 plasmid (Figure 1(e)).

### 3.2. *BCL6 Knockdown Inhibits *the Growth of Glioma Cell and Promotes Its Apoptosis

**Figure 2:**
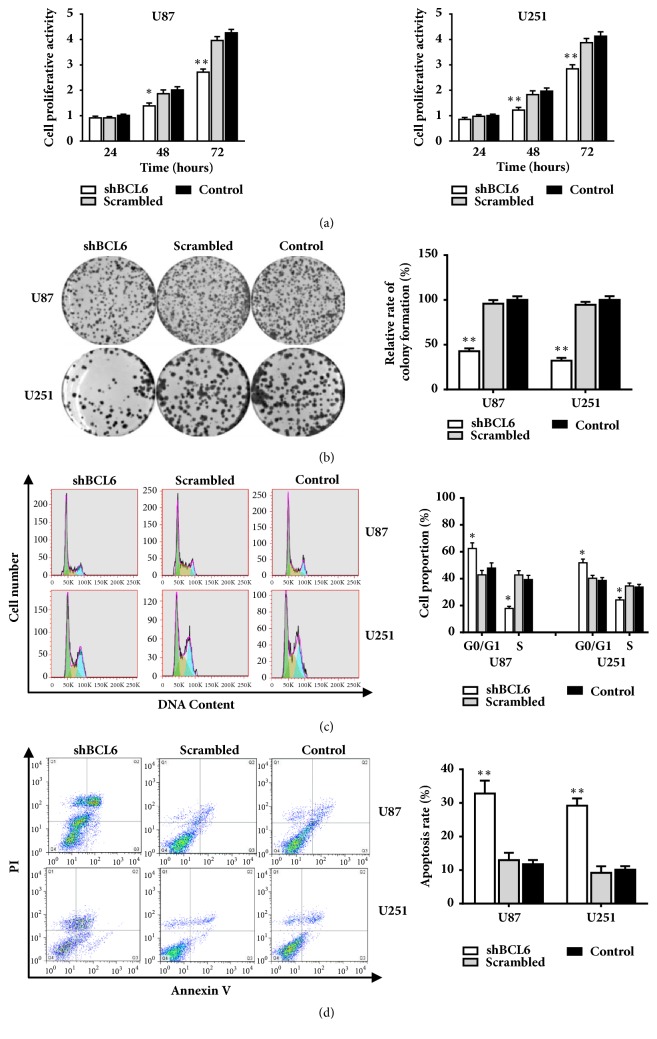
The influence of BCL6 on the proliferation capacity, cell cycle progression, and cell apoptosis rate of glioblastoma cells. (a) CCK8 assay detected that cell proliferation vitality was inhibited in glioblastoma cells after BCL6 knockdown in U87 and U251 cells. (b) The result of clone formation assay showed that cell proliferation capacity was reduced after BCL6 knockdown U87 and U251 cells. Relative expression was calculated with respect to the control group without treatment. ((c) and (d)) The result of flow cytometry analysis of cell cycle and apoptosis in U87 and U251 cells transfected with shBCL6 or scramble sequences (*∗∗* p<0.01; *∗* p<0.05).

We performed the cell viability assay and colony-formation assays to observe the effect of BCL6 on the glioma cell proliferation. The results showed that the growth of glioma cells transfected with shBCL6 was effectively inhibited compared with that of controls (Figures 2(a) and 2(b)).

As shown in the Figure 2(c), the downregulation of BCL6 reduced the proportion of cells in the S phase but elevated in the G0/G1 phase according to the measurement of FACS. These resulted suggested that BCL6 might play a critical role in G1-S transition in glioma. Then we stained the cells with Annexin V-FITC apoptosis detection kit after transfected 48 hours. The results showed that the depression of BCL6 induced the apoptosis of cells (Figure 2(d)).

### 3.3. BCL6 Promotes Cell Migration and Invasion In Vitro

**Figure 3:**
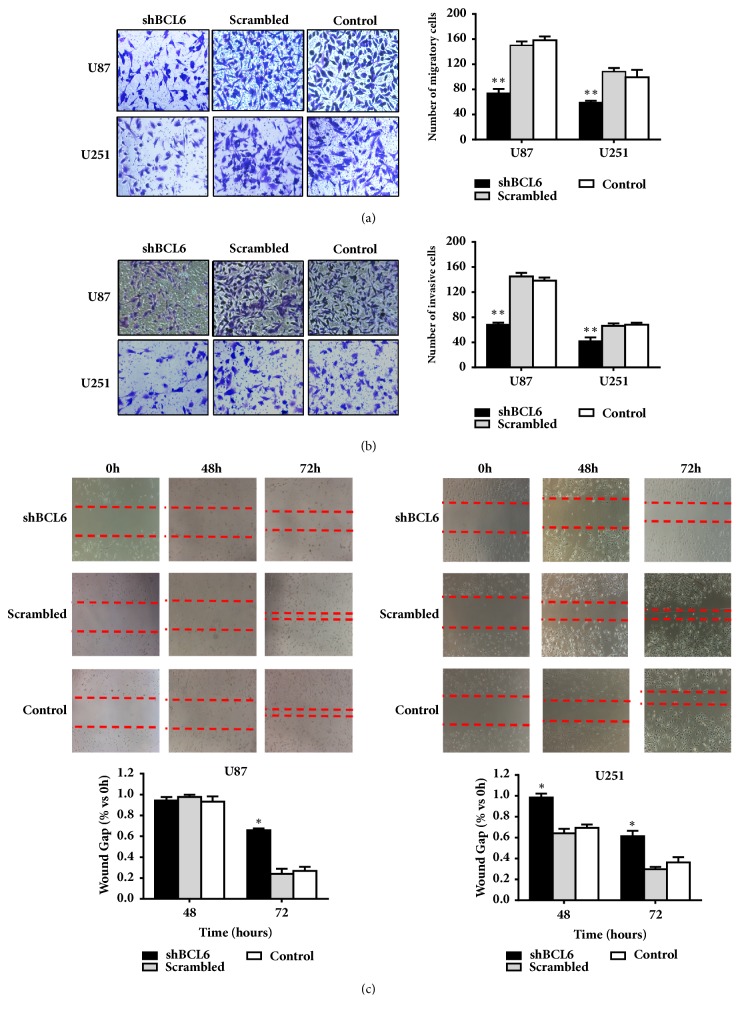
The influence of BCL6 on cell migration and invasion capacity. ((a) and (b)) Transwell assay was preformed to detect the migration and invasion capacity of glioblastoma cells after BCL6 knockdown. (c) Wound-healing assay was used to analyze the cell migration capacity of glioblastoma cells after BCL6 knockdown (*∗∗* p<0.01; *∗* p<0.05).

Tumor cells with BCL6 knockdown resulted in significant reduction of cell migration (Figure 3(a)) and invasion (Figure 3(b)) in Transwell assays. In contrast, control cells with nonspecific shRNA or without treatment displayed stronger capacity of migration and invasion through an extracellular matrix. The wound-healing assays showed that the U87 and U251 cells transfected with the shBCL6 plasmid closed the scratch slowly compared with the control cells (Figure 3(c)).

### 3.4. Knockdown of BCL6 Changes the Proteins Levels Related to Malignant Behaviors of Glioma Cells

**Figure 4:**
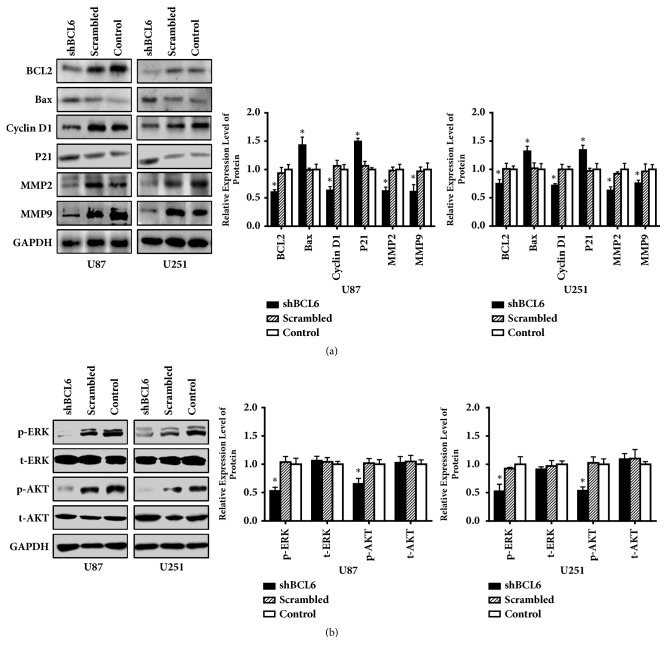
The influence of BCL6 on molecules related to malignant behaviors of glioblastoma cells. (a) The western blot results of BCL2, Bax, cyclin D1, P21, MMP2, and MMP9 in indicated cells. GAPDH was used as reference. (b) The protein levels of t-ERK, p-ERK, t-AKT, and p-AKT in indicated cells. Relative expression was calculated with respect to the control group without treatment (*∗* p<0.05).

Western blot was performed to investigate the effect of BCL6 on the malignant behavior of tumor cells in protein level. These related protein levels changed along with the knockdown of BCL6 (Figure 4(a)), which indicated that BCL6 might promote cell cycle progression, apoptosis, and metastasis.

To further investigate the mechanism related to BCL6 promoting malignant phenotype of glioma, we detected the protein level of t-ERK, p-ERK, t-AKT, and p-AKT. Although there was no significant difference in t-ERK and t-AKT level between the shBCL6 group and the control groups, the results showed that knockdown BCL6 could inhibit the phosphorylation of ERK and AKT (Figure 4(b)).

### 3.5. Knockdown of BCL6 Increases Chemosensitivity of Glioma Cells to Temozolomide

**Figure 5:**
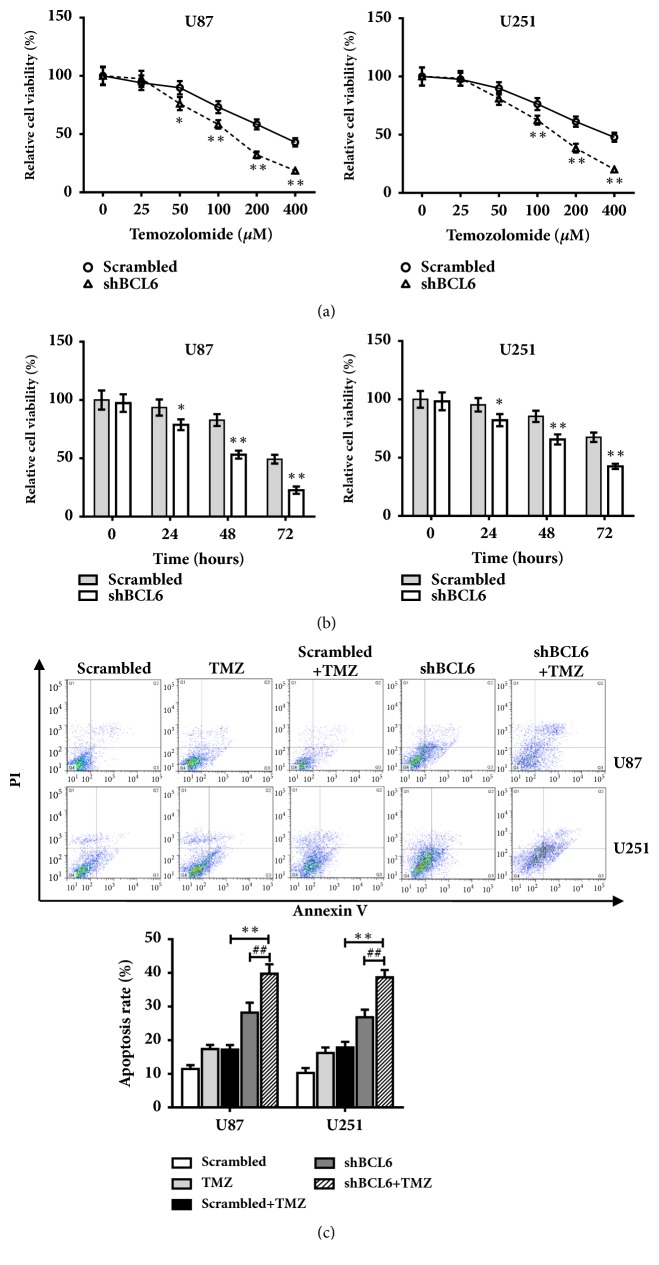
Reduction of BCL6 increases chemosensitivity of Glioma Cells to Temozolomide. (a) The cells transfected with scrambled shRNA or shBCL6 were treated with TMZ at different concentrations, and cell survival was measured by CCK8 assay. Data were normalized to the Scrambled group with 0 *μ*M TMZ. (b) The cell viability was assayed by CCK8 at 0, 24, 48, and 72 h after TMZ (150*μ*M). Data were normalized to the Scrambled group at 0 hours. (c) The apoptosis rate of U87 and U251 with different treatment was measured by flow cytometry assay (*∗∗* p<0.01; *∗* p<0.05; ^##^ p<0.01; ^#^ p<0.05).

The U87 and U251 cells were transfected with shBCL6 or scrambled and then treated with different concentrations of TMZ. As shown in Figure 5(a), reduction of BCL6 in U87 and U251 cells significantly increased chemosensitivity to TMZ treatment; the cell viability treated with shBCL6 and TMZ was significantly suppressed in a concentration-dependent manner. Furthermore, cell viability in the presence of TMZ (100 *μ*M) was assayed by CCK8 at different time points. The results showed that reduction of BCL6 significantly inhibited cell survival of both U87 and U251 cells in the presence of TMZ in a time dependent manner (Figure 5(b)). To test whether BCL6 may play a role in cell apoptosis in the presence of TMZ treatment, Annexin V level was detected by FCM. The results showed that the rate of apoptosis with combination treatment of shBCL6 + TMZ group was increased significantly (Figure 5(c)).

## 4. Discussion

As a human protooncogene, BCL6 was originally found in malignant lymphomas which acted as a regulator of the development and growth of B-lymphocyte [14–17]. Except for lymphomas, BCL6 also expresses high level in several cancer tissues, such as breast cancer [18], gastric cancer [6], ovarian cancer [7], non-small-cell lung cancer [19], and glioblastoma [12]. The overexpression of BCL6 usually results in malignant behavior of tumors above. However, it was related to a good survival rate in bladder carcinoma [20]. In our research, we further confirmed the high level of BCL6 was associated with a range of malignant characteristics in glioblastoma cells, including proliferation, migration, and invasion.

Currently, it is unclear that the reason for BCL6 upregulated in cancer tissues, the constitutive expression caused by translocation might partly explain the overexpression of this gene in cancer tissues [21, 22]. As translocation of BCL6 frequently occurred in high-grade glioblastoma [12], the expression of BCL6 had higher levels in Grades III and IV tumors than lower grade glioma tissues.

The function of BCL6 in cell proliferation and apoptosis has been explored for a long time with multiple molecules involved. Our research found that downregulation of BCL6 significantly reduced the expression of cyclin D1 and BCL2 while it upregulated the cyclin-dependent kinase inhibitor p21 and Bax. BCL6 was reported to induce the expression of cyclin D1 to cover the senescence response downstream of p53 by sequestering p21, but it could not repress p53-p19 pathway in senescence [23]. Considering that once BCL6 is deleted in glioma cells, there is not enough cyclin D1 to combine p21, it is not surprising that p21 expression is dependent on p53 in BCL6-depleted glioma cells [9]. Though BCL6 could suppress BCL2 in diffuse large B-cell lymphoma [24], we found that the expression of these two proteins exhibits positive correlation in glioblastoma cells. It was reported that BCL6 repressed the TP53 pathway in glioma [9], and p53 repressed BCL2 in some tumors [25]. Our research showed that p53 expression increased along with the knockdown of BCL6 in U87 cells. But in U251 cells (one of the p53-mutant cell lines), p53 expression had no significant changes. Besides, some research also showed that several molecules could upregulate BCL2 to inhibit cell apoptosis by AKT and ERK signaling pathways [26, 27] which was consistent with our result that knockdown of BCL6 inhibited the AKT and ERK pathways. In addition, BCL2 and Bax are key upstream regulatory factors of caspase cascade which involved in apoptosis [28], and regulation of BCL2/Bax expression alters proliferation and apoptosis in glioblastoma [29]. Moreover, BCL6 has a negative correlation with caspase-3 level in glioma tissues [12] and it targeting PDCD2 to activate the caspase cascade results in the inhibition of apoptosis in lymphomas [30]. Thus, we speculate that BCL6 inhibits apoptosis progress through several molecules to regulate BCL2/Bax to suppress the caspase cascade.

BCL6 exhibits its proinvasive ability in ovarian cancer [7] and breast cancer [8], and some molecules could perform their anticancer potential by suppressing the expression of BCL6 in ovarian cancer and breast cancer [13, 28, 29]. The expression level of BCL6 in ovarian cancer is correlated with the FIGO staging which is associated with the invasiveness of tumor. Moreover, several invasion-related proteins such as MMP2, MMP9, and N-Cadherin have positive correlation with BCL6 in ovarian tumor cells [7]. Similarly, the expression of BCL6 got intense along with the advance of glioma grades. And the proinvasive ability of this gene was further supported by the results of Transwell and wound-healing assays in our study. In addition, MMP2 and MMP9 were downregulated with the BCL6 knockdown. Matrix-metalloproteinase could degrade extracellular matrix proteins to overcome the physical barrier for glioblastoma cells, and studies showed that MMP2 and MMP9 could be regulated by several pathways such as Wnt and PI3K/AKT to promote GBM invasiveness [1].

The present research showed that knockdown BCL6 downregulated the expression of p-AKT and p-ERK. Both AKT and ERK are involved in receptor tyrosine kinase (RTK) pathway which is one of pathways emphasized in genomic analysis of GBM [31]. These two proteins participate in regulating many proteins involved in regulation of tumor malignant behaviors [2, 32]. Besides mentioned above, AKT is usually activated by phosphorylating at Thr308 and Ser473 sites, which is involved in regulation of multiple molecules to and promote cancer cell proliferation and migration as well as antiapoptosis in human cancers [33]. And p-ERK, a significant mediator of the cell cycle, could induce CDK complexes activation via upregulating the expression of cyclin D1, CDK4, and CDK6 as well as degrading CDK2/cyclin E inhibitor [2]. Moreover, previous studies showed that the effect BCL6 had on the activity of MEK-ERK and S6K-RPS6 cascades was partially mediated by targeting AXL [9]. Future studies may focus on the exact mechanism of BCL6 in AKT and MAPK pathways.

Furthermore, the glioblastoma was insensitive to chemotherapeutic drug, which led to chemotherapy failure and had a poor prognosis. In our study, we found that the reduction of BCL6 expression increased chemosensitivity of glioma cells to TMZ, and the apoptosis rates were upregulated in the reduction of BCL6 expression with TMZ group. Thus, BCL6 restoration approach may offer a new strategies and tactics to reverse or overcome multidrug resistance and hence to enhance the efficacy of TMZ treatment in glioma is one of the significant mission.

## 5. Conclusion

Our results show that BCL6 is expressed at a high level in glioma when compared with nontumor brain tissues. In addition, downregulation of BCL6 could inhibit proliferation, migration, and invasion, promote apoptosis, and induce cell cycle arrest via the AKT and MAPK pathway. Also, reduction of BCL6 increases chemosensitivity of glioblastoma to temozolomide. Thus, the identification of BCL6 may help us to understand the potential molecular mechanisms of the initiation and progression of glioma and provide us with a new prognostic marker for the management of glioma.

## Data Availability

The data used to support the findings of this study are available from the corresponding author upon request.

## References

[1] Paw I., Carpenter R. C., Watabe K., Debinski W., Lo H. W. (2015). Mechanisms regulating glioma invasion. *Cancer Letters*.

[2] Furnari F. B., Fenton T., Bachoo R. M. (2007). Malignant astrocytic glioma: genetics, biology, and paths to treatment. *Genes & Development*.

[3] Minniti G., de Sanctis V., Muni R. (2008). Radiotherapy plus concomitant and adjuvant temozolomide for glioblastoma in elderly patients. *Journal of Neuro-Oncology*.

[4] Basso K., Saito M., Sumazin P. (2010). Integrated biochemical and computational approach identifies BCL6 direct target genes controlling multiple pathways in normal germinal center B cells. *Blood*.

[5] Duan S., Cermak L., Pagan J. K. (2012). FBXO11 targets BCL6 for degradation and is inactivated in diffuse large B-cell lymphomas. *Nature*.

[6] Hirata Y., Ogasawara N., Sasaki M. (2009). BCL6 degradation caused by the interaction with the C-terminus of pro-HB-EGF induces cyclin D2 expression in gastric cancers. *British Journal of Cancer*.

[7] Wang Y. Q., Xu W. W., Wei P., Yang Y. S., Du X. (2015). BCL6 is a negative prognostic factor and exhibits pro-oncogenic activity in ovarian cancer. *American Journal of Cancer Research*.

[8] Yu J.-M., Sun W., Hua F. (2015). BCL6 induces EMT by promoting the ZEB1-mediated transcription repression of E-cadherin in breast cancer cells. *Cancer Letters*.

[9] Xu L., Chen Y., Dutra-Clarke M. (2017). CL6 promotes glioma and serves as a therapeutic target. *Proceedings of the National Academy of Sciences of the United States of America*.

[10] Wu Q., Liu X., Yan H. (2014). B-cell lymphoma 6 protein stimulates oncogenicity of human breast cancer cells. *BMC Cancer*.

[11] Bos R., van Diest P. J., van der Groep P. (2003). Protein expression of B-cell lymphoma gene 6 (BCL-6) in invasive breast cancer is associated with cyclin D_1_ and hypoxia-inducible factor-1_*α*_ (HIF-1_*α*_). *Oncogene*.

[12] Ruggieri S., Tamma R., Marzullo A. (2014). Translocation of the proto-oncogene Bcl-6 in human glioblastoma multiforme. *Cancer Letters*.

[13] Shan W., Li J., Bai Y., Lu X. (2016). miR-339-5p inhibits migration and invasion in ovarian cancer cell lines by targeting NACC1 and BCL6. *Tumor Biology*.

[14] Ci W., Polo J. M., Cerchietti L. (2009). The BCL6 transcriptional program features repression of multiple oncogenes in primary B cells and is deregulated in DLBCL. *Blood*.

[15] Thieblemont C., Brière J. (2013). MYC, BCL2, BCL6 in DLBCL: impact for clinics in the future?. *Blood*.

[16] Phan R. T., Dalla-Favera R. (2004). The BCL6 proto-oncogene suppresses p53 expression in germinal-centre B-cells. *Nature*.

[17] Polo J. M., Dell'Oso T., Ranuncolo S. M. (2004). Specific peptide interference reveals BCL6 transcriptional and oncogenic mechanisms in B-cell lymphoma cells. *Nature Medicine*.

[18] Walker S. R., Liu S., Xiang M. (2015). The transcriptional modulator BCL6 as a molecular target for breast cancer therapy. *Oncogene*.

[19] Sun C., Li S., Yang C. (2016). MicroRNA-187-3p mitigates non-small cell lung cancer (NSCLC) development through down-regulation of BCL6. *Biochemical Biophysical Research Communications*.

[20] Bahria-Sediki I. B., Yousfi N., Paul C. (2016). Clinical significance of T-bet, GATA-3, and Bcl-6 transcription factor expression in bladder carcinoma. *Journal of Translational Medicine*.

[21] Ye B. H., Lista F., Lo Coco F. (1993). Alterations of a zinc finger-encoding gene, BCL-6, in diffuse large-cell lymphoma. *Science*.

[22] Kuukasjärvi T., Karhu R., Tanner M. (1997). Genetic heterogeneity and clonal evolution underlying development of asynchronous metastasis in human breast cancer. *Cancer Research*.

[23] Shvarts A., Brummelkamp T. R., Scheeren F. (2002). A senescence rescue screen identifies BCL6 as an inhibitor of anti-proliferative p19ARF-p53 signaling. *Genes & Development*.

[24] Saito M., Novak U., Piovan E. (2009). BCL6 suppression of BCL2 via Miz1 and its disruption in diffuse large B cell lymphoma. *Proceedings of the National Acadamy of Sciences of the United States of America*.

[25] Wu Y.-L., Mehew J. W., Heckman C. A., Arcinas M., Boxer L. M. (2001). Negative regulation of bcl-2 expression by p53 in hematopoietic cells. *Oncogene*.

[26] Zhang B., Liu Y., Li Y., Zhe X., Zhang S., Zhang L. (2018). Neuroglobin promotes the proliferation and suppresses the apoptosis of glioma cells by activating the PI3K/AKT pathway. *Molecular Medicine Reports*.

[27] Liu J., Li Q., Liu Z. (2016). Harmine induces cell cycle arrest and mitochondrial pathway-mediated cellular apoptosis in SW620 cells via inhibition of the Akt and ERK signaling pathways. *Oncology Reports*.

[28] Adams J. M., Cory S. (2007). The Bcl-2 apoptotic switch in cancer development and therapy. *Oncogene*.

[29] Skała E., Sitarek P., Toma M. (2016). Inhibition of human glioma cell proliferation by altered Bax/Bcl-2-p53 expression and apoptosis induction by *Rhaponticum carthamoides* extracts from transformed and normal roots. *Journal of Pharmacy and Pharmacology*.

[30] Baron B. W., Hyjek E., Gladstone B., Thirman M. J., Baron J. M. (2010). PDCD2, a protein whose expression is repressed by BCL6, induces apoptosis in human cells by activation of the caspase cascade. *Blood Cells, Molecules, and Diseases*.

[31] Brennan C. W., Verhaak R. G. W., McKenna A. (2013). The somatic genomic landscape of glioblastoma. *Cell*.

[32] Tran T. H., Utama F. E., Lin J. (2010). Prolactin inhibits BCL6 expression in breast cancer through a stat5a-dependent mechanism. *Cancer Research*.

[33] Wang L., Ouyang F., Liu X. (2016). Overexpressed CISD2 has prognostic value in human gastric cancer and promotes gastric cancer cell proliferation and tumorigenesis via AKT signaling pathway. *Oncotarget *.

